# Image-guided interstitial brachytherapy boost for keratinizing squamous cell carcinoma of inferior wall of the nasopharynx

**DOI:** 10.1259/bjrcr.20200005

**Published:** 2020-08-21

**Authors:** Naoya Murakami, Seiichi Yoshimoto, Masakazu Uematsu, Tairo Kashihara, Kana Takahashi, Koji Inaba, Kae Okuma, Hiroshi Igaki, Yuko Nakayama, Koji Masui, Ken Yoshida, Jun Itami

**Affiliations:** 1 Department of Radiation Oncology, National Cancer Center Hospital, 5-1-1 Tsukiji, Chuo-ku, Tokyo, Japan; 2 Department of Head and Neck Surgery, National Cancer Center Hospital, 5-1-1 Tsukiji, Chuo-ku, Tokyo, Japan; 3 Department of Radiology, Kyoto Prefectural University of Medicine, Kyoto, Japan; 4 Department of Radiation Oncology, Osaka Medical College, Takatsuki, Japan

## Abstract

High-dose-rate interstitial brachytherapy (HDR-ISBT) is relatively rarely applied for the head and neck cancer because of its anatomical complexity and difficulty of applicator placement. However, its dose distribution is more confined even better than intensity-modulated radiation therapy (IMRT) and can deliver a higher dose while sparing surrounding normal tissues. In this case report, the effectiveness of HDR-ISBT as a boost following IMRT for keratinizing squamous cell carcinoma of nasopharynx was presented. A 76-year-old female who suffered from cT3N0M0 keratinizing squamous cell carcinoma of the nasopharynx was treated with definitive concurrent chemoradiation therapy involving IMRT. However, physical examination and laryngoscope fibre finding showed evident residual tumour at 60 Gy of IMRT, then, boost HDR-ISBT was proposed. After delivering 66 Gy of IMRT, CT image-guided HDR-ISBT 4 Gy in a single fraction was performed under local anaesthesia and sedation. MRI taken 5 months after HDR-ISBT showed remarkable shrinkage of the primary tumour. After HDR-ISBT, the remaining session of IMRT was delivered from the next day until 70 Gy in 35 fractions. It was demonstrated that boost HDR-ISBT combined with IMRT for keratinizing squamous cell carcinoma of the nasopharynx was performed safely and showed favourable efficacy.

## Introduction

Because of its anatomical complexity, it is generally difficult to safely insert interstitial brachytherapy applicators in the head and neck region. Therefore, brachytherapy is rarely used in the head and neck region except early-stage tongue cancer^1–7^ or other oral cavity cancers.^8–11^ It is true that intracavitary brachytherapy (ICBT) is used for nasopharyngeal cancer (NPC),^12–15^ because in ICBT, the source is located in the nasopharyngeal cavity, it is usually difficult to deliver adequate dose deeper than 5 mm from the surface of the cavity.

The vast majority of NPC consists of non-keratinizing squamous cell carcinoma (NKSCC) and it is well-known that carcinogenesis of NKSCC is related to Epstein-Barr virus (EBV) infection.^16,17^ While NKSCC responds well to radiation therapy or chemotherapy, keratinizing squamous cell carcinoma (KSCC) respond poorly to such treatments and is associated with worse clinical outcomes.^18,19^ In our single institutional retrospective study, it was also shown that 3-year locoregional control for patients with KSCC and NKSCC were 25 and 92%, respectively (*p* < 0.001, HR 16.045 (95%CI 3.181–80.931).^20^

In this case report, authors successfully applied CT image-guided high-dose-rate interstitial brachytherapy (HDR-ISBT) as a boost for poorly responding NPC with KSCC subtype. Written informed consent was obtained from the patients and this case report was approved by the Institutional Review Board of National Cancer Center Hospital (the approved number is 2017–091) according to the ethical standards laid down in the Declaration of Helsinki.

## Clinical presentation

**Figure 1.**
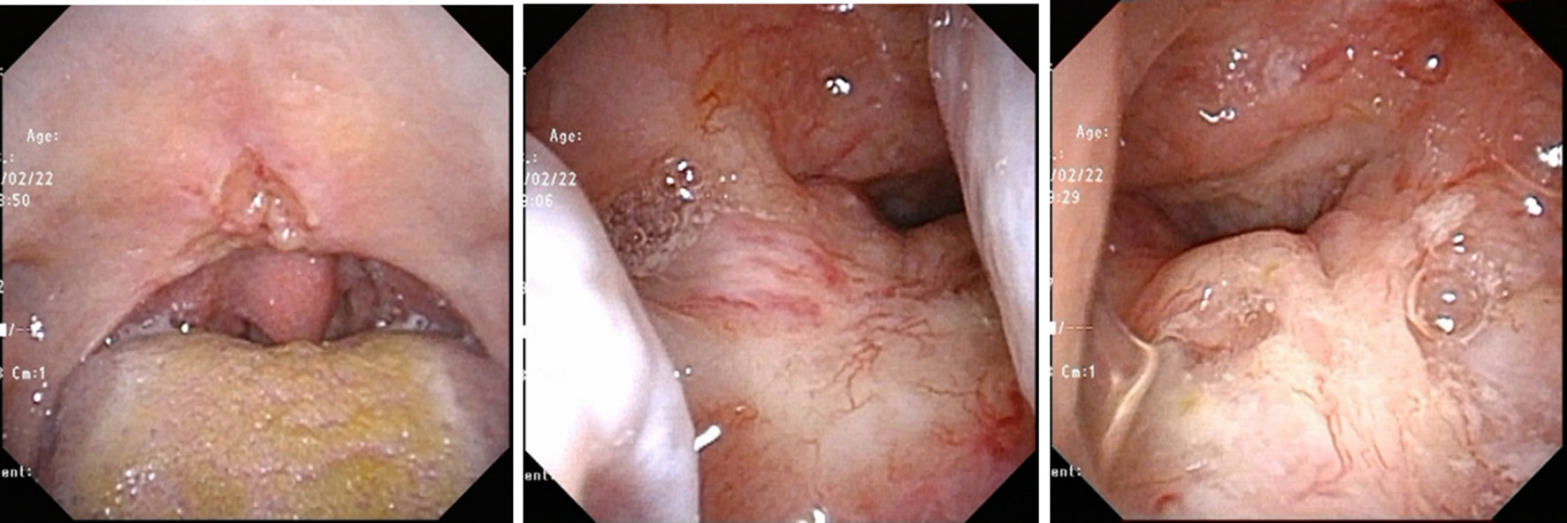
Laryngofiberscopic findings of nasopharyngeal cancer before treatment. The main tumour was located in the inferior wall of the nasopharynx which can be seen per-orally.

**Figure 2.**
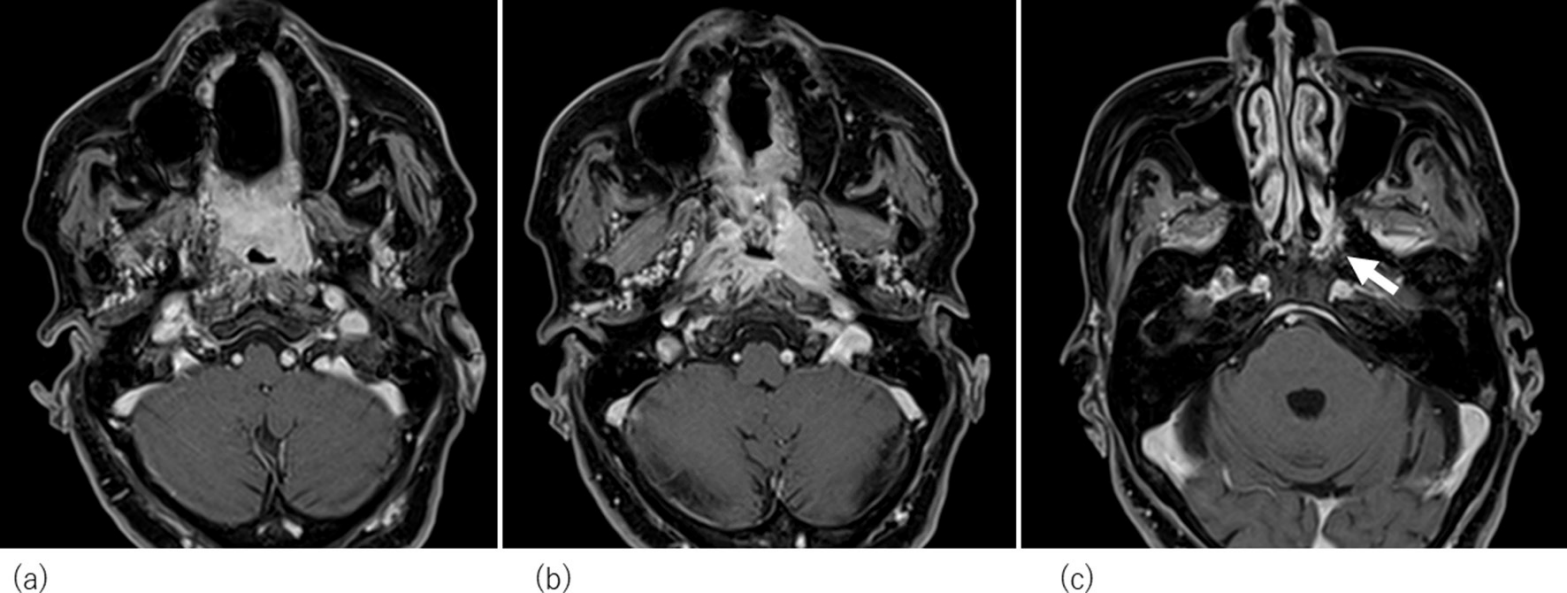
Magnetic resonance images of nasopharyngeal squamous cell carcinoma in the inferior wall of the nasopharynx before treatment. Because left sphenoid bone invasion was found (white arrow in Figure 2 (cFigure)), the tumour was classified as T3.

**Figure 3.**
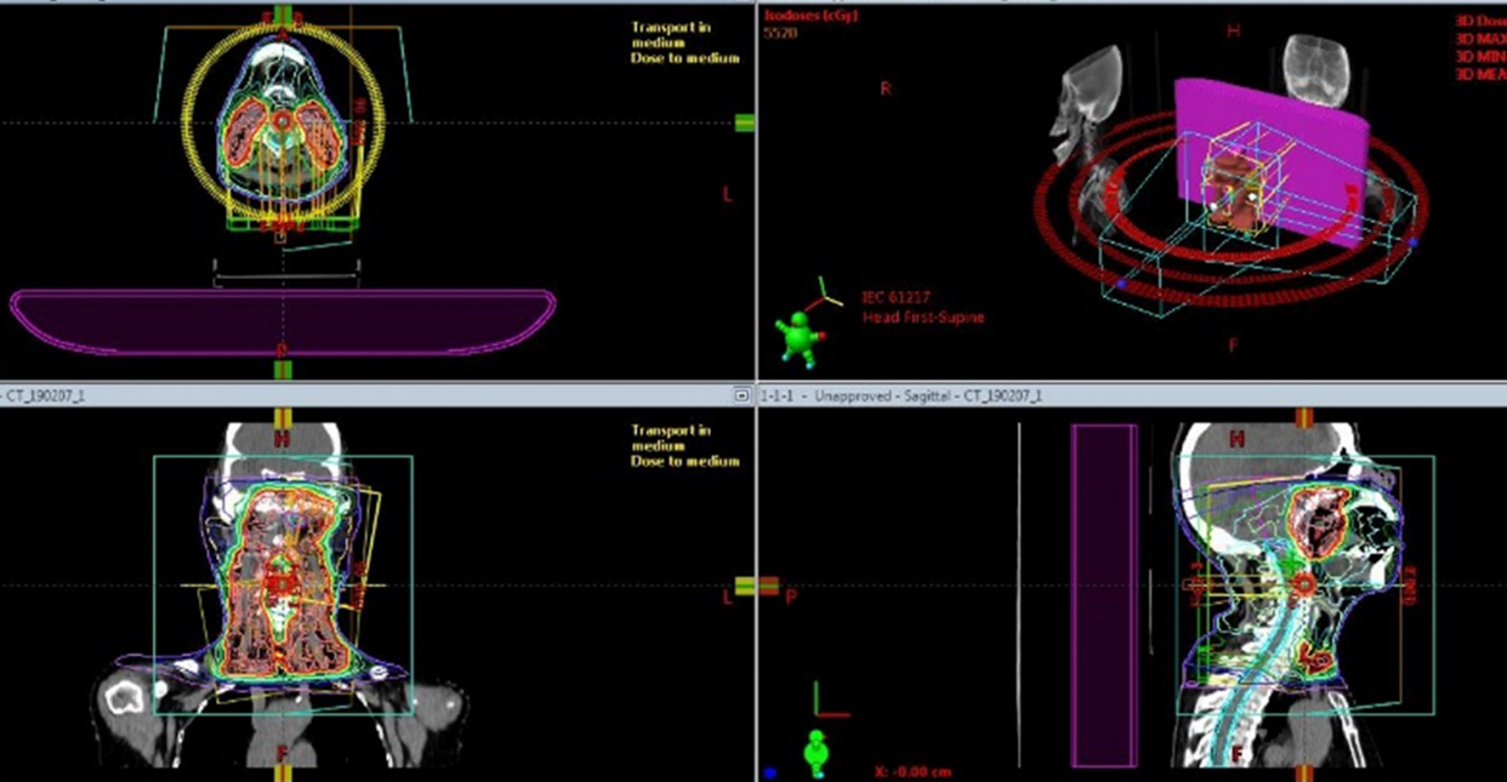
Figure 3 shows a dose distribution of intensity modulated radiation therapy for nasopharyngeal cancer.

**Figure 4.**
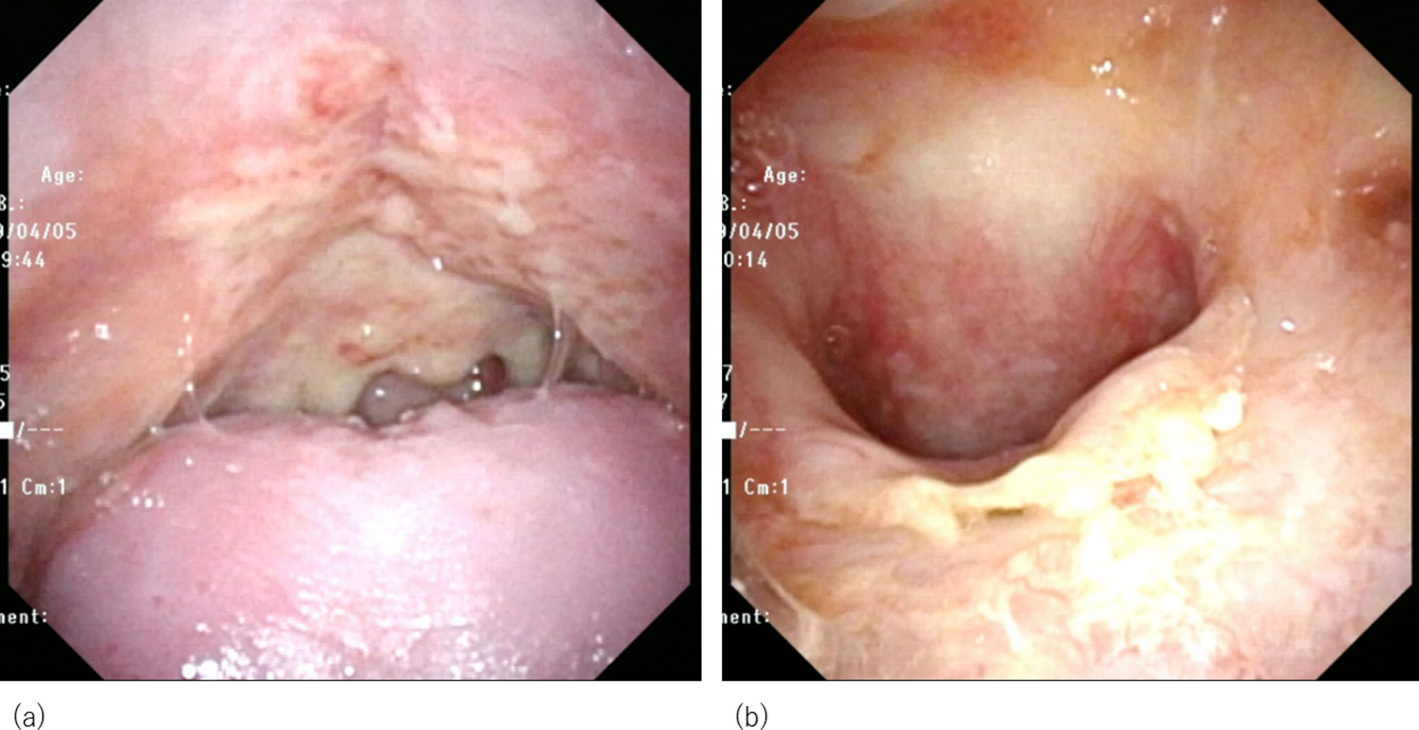
Laryngofiberscopic findings at 60 Gy of external beam radiation therapy. Even after 60 Gy, thick tumour still could be seen in the inferior wall of the nasopharynx and a hard tumour could still be palpable.

**Figure 5.**
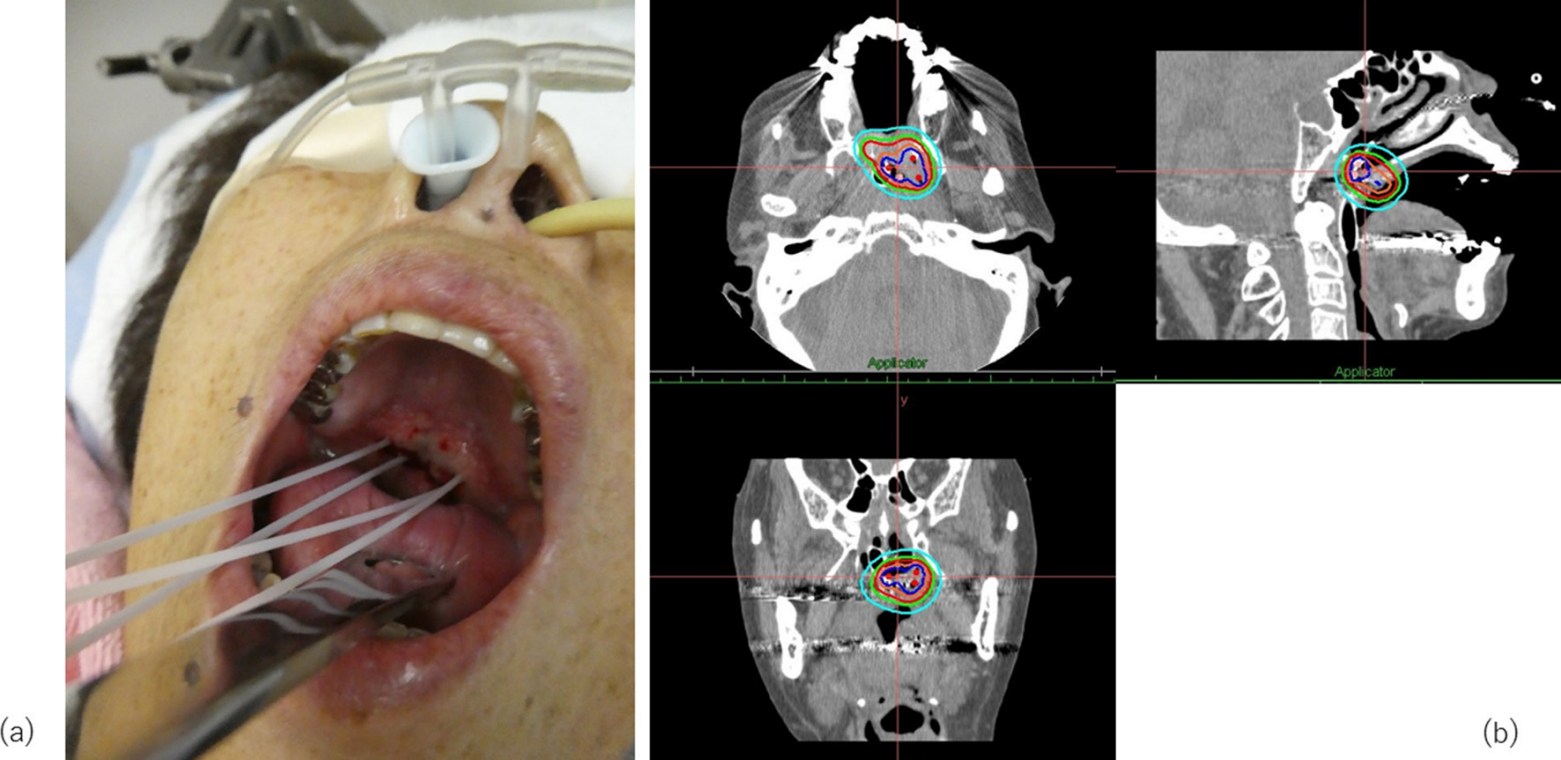
(a) Under local anesthesia and sedation, four 5 French ProGuide® sharp plastic needles (Nucletron, an Elekta company, Elekta AB, Stockholm, Sweden) were inserted transorally through the tumour. Depth of the needles was determined by CT image. (b) Isodose distribution of the interstitial implant with the red and blue line representing the 100 and 200% isodose, respectively.

A 76 year-old-female, who had a 7.5 pack-year smoking history, suffered from clinically T3N0M0 keratinizing squamous cell carcinoma of the nasopharynx. While most NPCs develop from the fossa of Rosenmüller or the posterior wall, the main tumour was located in the inferior wall of the nasopharynx (the superior surface of the soft palate) penetrating the soft palate which can be visual per-orally (Figure 1). The tumour extended to the left sidewall of the nasopharynx. Pretreatment MRI pointed out its slight cranial extension into left sphenoid bone (Figure 2), then it was diagnosed as T3. Histopathologic analysis of the pretreatment biopsy specimen showed KSCC subtype with negative for EBER-ISH. p16 status of the tumour was also investigated and it was found to be negative. Serum EBV-DNA was also negative. According to the standard treatment for NPC, concurrent chemoradiation (CCRT) was performed. Radiation therapy was delivered by conventional 2 Gy per fraction with volumetric modulated arc therapy (Figure 3) concurrent with three cycles of tri-weekly cisplatin (80 mg/m^2^). The tumour response was assessed weekly by physical examination and a flexible laryngofiberscope. Unlike usual NPC with NKSCC subtype, the tumour responded poorly to the CCRT; still a thick tumour could be seen in the inferior wall of the nasopharynx and a hard tumour could still be palpable even after 60 Gy was delivered (Figure 4). At this time, it was anticipated that tumour control could not be obtained only by 70 Gy of external beam radiation therapy. Since the main portion of the tumour was located in the inferior wall of the nasopharynx which consists the superior surface of the soft palate where no dangerous anatomical structures such as internal carotid artery or nerve exist. HDR-ISBT boost was offered to the patient. Because this patient received nutritional support by means of gastrostomy, she only had mild mucositis in the soft palate and it was supposed that she could tolerate additional HDR-ISBT intercalated with the external beam radiation therapy (EBRT). A single fraction of HDR-ISBT boost was inserted after 64 Gy of VMAT and the rest of VMAT were continued from the next day of HDR-ISBT until 70 Gy; therefore, a total of 74 Gy was given in 52 days (7.4 weeks). HDR interstitial needle insertion was performed on the simulation CT table. Under local anaesthesia and sedation, four 5 French ProGuide® sharp plastic needles (Nucletron, an Elekta company, Elekta AB, Stockholm, Sweden) were inserted trans-orally under flexible laryngofiberscope and CT guidance (Figure 5A and B). CTV at the time of HDR-ISBT included inferior and left sidewall of the nasopharynx; because of its difficulty in inserting needles, the sphenoid bone extension was not covered by HDR-ISBT. Dose calculation was performed using Oncentra Brachy v.4.5.1 (Nucletron, an Elekta company, Elekta AB, Stockholm, Sweden) based on the CT image (image-guided brachytherapy) (Figure 5). Dose calculation was performed so that the 100% prescribed isodose line of 4 Gy covered the CTV while avoiding the hyper-dose-sleeve, representing 200% of the prescribed reference dose, as small as possible. Because patterns of failure for NPC with KSCC subtype is generally locoregional, in contrast to NPC with NKSCC whose dominant failure pattern is distant, no adjuvant chemotherapy (the combination of cisplatin and 5-fluorouracil which is given one month after finish of CCRT) was administered to the patient.

**Figure 6.**
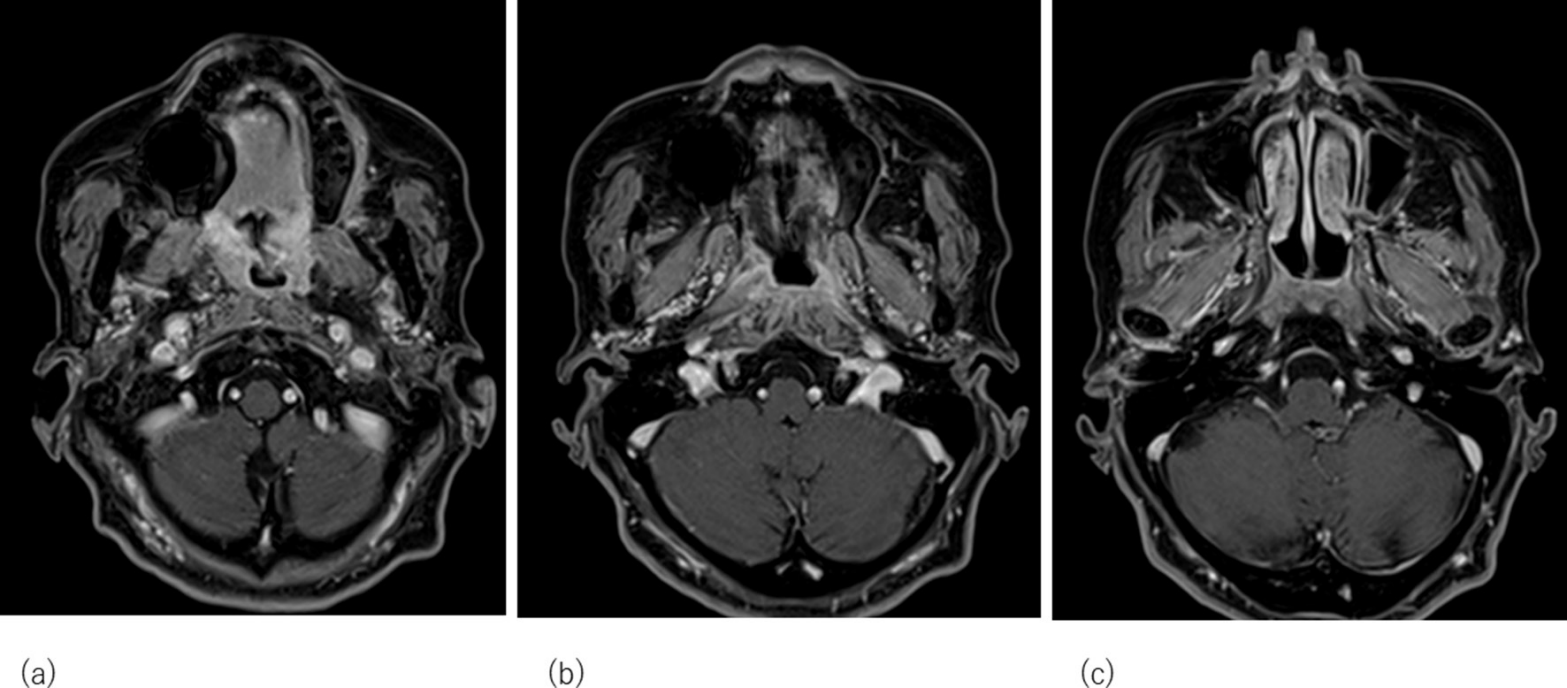
Magnetic resonance image taken 5 months after interstitial brachytherapy. No evident residual tumour was found in the nasopharynx.

MRI taken 2 and 5 months after completion of the combination of VMAT and HDR-ISBT showed marked shrunk of the primary disease with increased apparent diffusion coefficient value in diffusion-weighted imaging, suggesting a decrease in tumour viability (Figure 6).

## Discussion

For those NPCs that respond poorly to CCRT, ICBT could be an option for boosting local dose after EBRT.^12–14^ However, since the source is located in the nasopharyngeal cavity in ICBT, it is generally difficult to deliver an adequate dose deeper than 5 mm from the surface of the cavity; otherwise, a huge dose would be delivered to the surface of the nasopharyngeal mucosa which could result in devastating consequences. In the management of head and neck cancer, ISBT is used with or without a combination of EBRT. The most commonly used site is oral cavity^1–3,5–8,10,11^ followed by oropharyngeal cancer.^4,9,21–24^ ISBT is also used in the recurrent cases.^25–28^ Recently, there are several reports regarding ISBT for NPC.^29–32^ However, these tumours were located mainly on posterior or lateral part of the nasopharynx. In the current case, because the mail tumour was located in the inferior wall where no dangerous anatomic structure exists, it was possible to insert interstitial needles without causing any severe complications. A single dose of 4 Gy was selected in the current case, because several guidelines for head and neck brachytherapy recommended a dose per fraction between 2.5 and 6 Gy.^33–36^ However, optimal dose per fraction for NPC boost HDR-ISBT should be investigated in the following future studies. To the best of our knowledge, this is a first report concerning with HDR-ISBT for inferior wall of the NPC. Co LA et al reported in a systematic review and meta-analysis^37^ that dose-escalation for NPC patients were only beneficial if tumours were T1-2 and patients were treated by RT alone. However, in this study, ICBT was mostly applied as a modality to deliver additional dose after EBRT; therefore, the benefit of giving additional dose for NPC patients by means of ISBT is unknown and should be investigated in the future studies. The limitation of this case report is that not all pretreatment gross tumour volume (GTV) was encompassed by HDR-ISBT, but only GTV after EBRT was covered. Therefore, further careful follow-up is needed to see the long-term efficacy of boost HDR-ISBT for NPC.

In this case report, a KSCC subtype NPC patient with an additional dose given by means of image-guided HDR-ISBT after EBRT was presented. For persistent tumours against conventional EBRT, it could be an alternative option to give additional dose by HDR-ISBT even in patients with NPC.

## Learning points

Although intracavitary brachytherapy long has been performed for nasopharyngeal cancer, interstitial brachytherapy is rarely performed for nasopharyngeal cancer. However, if it is possible to insert needles to the residual tumour after external beam radiation therapy, it was shown that interstitial brachytherapy boost could be given even for nasopharyngeal cancer.
